# *Lactiplantibacillus plantarum* as a Psychobiotic Strategy Targeting Parkinson’s Disease: A Review and Mechanistic Insights

**DOI:** 10.3390/nu17193047

**Published:** 2025-09-24

**Authors:** Wu-Lin Chen, Fu-Sheng Deng, Ying-Chieh Tsai

**Affiliations:** 1 Research and Development Department, Bened Biomedical Co., Ltd., Taipei 115011, Taiwan; wlchen89@benedbiomed.com (W.-L.C.); stephen@benedbiomed.com (F.-S.D.); 2 Institute of Biochemistry and Molecular Biology, National Yang Ming Chiao Tung University, Taipei 11221, Taiwan

**Keywords:** Parkinson’s disease, *Lactiplantibacillus plantarum*, microbiota-gut–brain axis (MGBA), psychobiotics

## Abstract

Parkinson’s disease (PD) is a progressive neurodegenerative disorder characterized by the pathological aggregation of α-synuclein (α-syn), the loss of dopaminergic neurons, and the appearance of both motor and non-motor symptoms. Emerging evidence suggests a bidirectional influence of the microbiota–gut–brain axis in PD pathogenesis, where gut dysbiosis contributes to increased intestinal barrier permeability, immune activation, chronic inflammation, oxidative stress, α-syn misfolding, and neurotransmitter imbalance. These findings are increasing interest in probiotics as microbiota-targeted interventions that restore intestinal and systemic homeostasis. *Lactiplantibacillus plantarum*, a probiotic species with remarkable environmental adaptability and genomic plasticity, has emerged as a promising candidate for PD management. Preclinical studies demonstrate that specific *Lpb. plantarum* strains, such as PS128 or CCFM405, can beneficially modulate gut microbial communities, reinforce barrier integrity, regulate bile acid metabolism, attenuate neuroinflammatory responses, and improve motor deficits in PD-like mice. In addition, *Lpb. plantarum* DP189 or SG5 interventions can significantly reduce α-syn aggregation in the brain via suppression of oxidative stress, modulation of neuroinflammatory responses, and activation of neurotrophic factors. Recent evidence even suggests that *Lpb. plantarum*-derived extracellular vesicles may possess anti-PD activity by influencing host gene expression, neuronal function, and immune modulation. Although robust clinical data are still limited, preliminary clinical trials indicate that supplementation with PS128 or certain *Lpb. plantarum*-contained consortiums can alleviate constipation, improve gastrointestinal function, reduce systemic inflammation, and even ameliorate motor symptoms when used alongside standard dopaminergic therapies. In this review, we provide an integrated overview of preclinical, clinical, and mechanistic insights, and evaluate the translational potential of *Lpb. plantarum* as a safe and diet-based strategy to target the microbiota-gut–brain axis in PD.

## 1. Introduction

### 1.1. Overview of Parkinson’s Disease: Epidemiology, Clinical Features, and Current Therapeutic Challenges

Parkinson’s disease (PD) is the second most common neurodegenerative disorder after Alzheimer’s disease, and is widely regarded as the most prevalent movement disorder [1]. According to the Global Burden of Disease Study 2021, approximately 11.77 million people worldwide had PD, and the number of individuals affected globally is projected to exceed 18 million by 2035 [2]. This rapid increase indicates the urgency to develop novel therapeutic strategies and signals a parallel rise in the economic burden imposed by PD. In the United States, direct healthcare costs related to PD were estimated at $51.9 billion in 2017, escalating to $79.1 billion in 2037 when considering the total disability burden in advanced PD [3]. These numbers reflect the profound impact of PD not only on patients and their families but also on healthcare systems worldwide.

PD is a progressive neurodegenerative disorder that is primarily characterized by motor deficits, including resting tremors, bradykinesia, rigidity, and postural instability [4]. As the disease progresses, additional motor impairments such as gait disorders, freezing of gait, falls, and dysphagia may emerge [5]. In fact, a range of non-motor symptoms, including olfactory dysfunction, sleep behavior disorders, depression, and gastrointestinal (GI) disturbances, often appear years before the onset of motor deficits [6,7]. The early appearance of non-motor symptoms in PD suggests that pathological processes may be initiated in the peripheral nervous system before the onset of central nervous system (CNS) involvement [8]. These non-motor symptoms may serve as an early detection signal and support the perspective that PD is a systemic neurodegenerative disorder rather than being confined solely to the motor system [6,9].

Mechanistically, the motor deficits of PD are caused by the progressive degeneration of dopaminergic neurons in the substantia nigra pars compacta (SNpc), tightly associated with the misfolding and aggregation of α-synuclein (α-syn) [10]. This neuronal loss leads to a substantial reduction in striatal dopamine levels, which subsequently disrupts basal ganglia circuitry and impairs the initiation and regulation of voluntary movements [11]. Currently, drug treatment is a major option for PD, focusing on the symptomatic relief of motor deficits [12,13]. Levodopa (L-DOPA), the metabolic precursor of dopamine, is the most effective and widely used medication because of its ability to cross the blood–brain barrier (BBB) and replenish striatal dopamine levels [14]. To enhance drug efficacy, L-DOPA is often used in combination with catechol-O-methyltransferase or monoamine oxidase-B inhibitors, which can extend the half-life of L-DOPA and prevent peripheral dopamine degradation [14,15]. Additionally, dopamine receptor agonists, which directly stimulate the dopamine receptors, are used either as monotherapy or in combination with L-DOPA to improve symptoms [16]. However, these treatments are primarily symptomatic and do not alter disease progression. Prolonged use of L-DOPA is even associated with motor complications, such as L-DOPA-induced dyskinesia, dystonia, and ON/OFF fluctuations in motor performance [17].

Given the limitations of current therapies, there is a growing interest in complementary or alternative treatments that not only alleviate PD motor or non-motor symptoms but also reduce drug-related side effects. In this framework, probiotics, particularly psychobiotics, have emerged as a promising and feasible adjunctive strategy. Although probiotics now cannot fully replace pharmaceutical treatments, some functional species are able to modulate gut microbiota composition, influence the microbiota-gut–brain axis (MGBA) function, and potentially intervene in the mechanisms underlying neurodegeneration [18]. These findings make probiotics a viable option for PD patients.

### 1.2. Emergence of the Microbiota–Gut–Brain Axis and the Therapeutic Potential of Lactiplantibacillus plantarum on Parkinson’s Disease

In recent years, accumulating research on the MGBA has highlighted the potential involvement of gut microbiota in the initiation and progression of PD [19,20]. MGBA is a complex bidirectional communication network that integrates the CNS, enteric nervous system (ENS), immune system, metabolic pathways, and gut microbiota [21,22]. Mounting evidence indicates that alterations in gut microbiota composition, commonly referred to as gut dysbiosis, may contribute to PD pathophysiology through multiple interrelated mechanisms, including modulation of neuroinflammation, induction of oxidative stress, disruption of short-chain fatty acid (SCFA) production, impairment of intestinal barrier integrity, increased BBB permeability, and protein misfolding and aggregation [23,24,25]. In addition to the local effects on the GI tract, gut dysbiosis may induce systemic disturbances that compromise nervous system function. Disruptions in the gut microbial balance have been shown to trigger systemic inflammation, impair peripheral immune regulation, and promote the production of neurotoxic metabolites, ultimately causing neuronal injury [26].

Psychobiotics, a type of probiotics, have attracted increasing attention as potential adjunctive interventions capable of modulating MGBA interactions and alleviating PD processes [18,27]. This term was first proposed by Dinan and colleagues (2013) and is defined as “a live organism that, when ingested in adequate amounts, produces a health benefit in patients suffering from psychiatric illness” [28]. Psychobiotics may provide therapeutic benefits by restoring the microbial balance and enhancing the functional activity of beneficial bacteria [18,27]. For example, the most widely studied bacteria for their psychobiotic effects belong to the genus *Lactobacillus* and *Bifidobacterium*, such as *Lactiplantibacillus plantarum*, *Lacticaseibacillus casei*, *Bifidobacterium lactis*, and *B. longum* [27]. These strains have been reported to correct gut permeability and restore gut microbial balance, leading to attenuation of intestinal inflammation, reinforcement of gut barrier integrity, modulation of immune responses, reduction in oxidative stress, and regulation of neurotransmitter metabolism [18,27,29,30,31]. Collectively, these effects may directly or indirectly contribute to neuronal protection and potentially slow the progression of PD.

Among various psychobiotic species, *Lpb. plantarum* has emerged as one of the most extensively studied candidates owing to its robust environmental adaptability, broad-spectrum metabolic capacity, and ability to produce various bioactive compounds [32,33]. Importantly, several preclinical studies suggest that some of the *Lpb. plantarum*-specific strains may modulate gut microbiota composition, inhibit neuroinflammatory signaling, stabilize intracellular calcium homeostasis, regulate gut-derived metabolites, and potentially interfere with α-syn aggregation [32,34,35,36,37,38]. These fundings make *Lpb. plantarum* a highly promising candidate for further investigation into PD-related therapeutic applications.

In this review, we aimed to explore the psychobiotic potential of *Lpb. plantarum* strains as gut-targeted therapeutic candidates for PD. We focused on the mechanisms underlying dopaminergic neuroprotection, with an MGBA-centered perspective, highlighting strains that have preclinical and clinical support for improving both motor and non-motor symptoms of PD.

## 2. Preclinical Evidence Supporting the Functional Role of *Lactiplantibacillus plantarum* in Parkinson’s Disease

### 2.1. Preclinical Animal Models for Elucidating Microbiota-Gut–Brain Axis Mechanisms in Parkinson’s Disease

**Figure 1:**
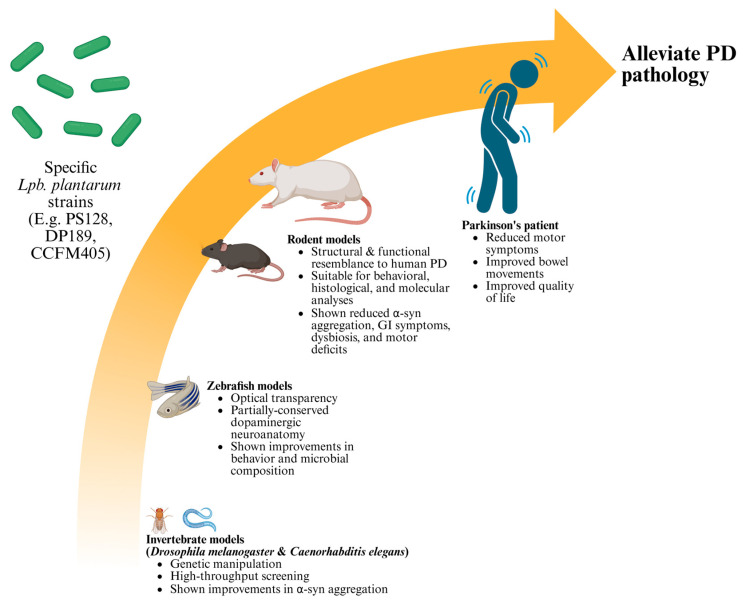
Specific *Lpb. plantarum* strains show anti-PD activity in animal models and patients.

Animal models remain indispensable for PD research owing to the limited accessibility of human tissues and the invasiveness of clinical sampling [39,40,41]. These models allow the systematic investigation of PD pathogenesis and provide a controlled platform for testing novel therapeutic agents, including dietary and probiotic interventions [42,43,44]. Given the multifactorial etiology of PD, which includes genetic predisposition, environmental toxins, mitochondrial dysfunction, and neuroinflammation, various model organisms have been developed to reproduce specific pathological features [13,42]. As shown in Figure 1, invertebrate models such as *Caenorhabditis elegans* and *Drosophila melanogaster* are widely used for genetic manipulation and high-throughput screening [45,46,47]. Nevertheless, their relatively simple nervous system, lack of endogenous α-syn expression, and limited behavioral repertoire restrict their translational applicability in recapitulating the complex pathophysiology of human PD [48,49,50]. Zebrafish serve as an intermediate vertebrate model with optical transparency and a partially conserved dopaminergic neuroanatomy [51,52,53]. However, their small size, relatively limited repertoire of complex motor coordination, and limited availability of validated antibodies for certain neural and immune markers may constrain their applicability in long-term neurobehavioral and immunological investigations [51,54]. In contrast, rodent models are most widely employed because of their structural and functional resemblance to the human nigrostriatal system and their suitability for behavioral, histological, and molecular analyses [13,55,56].

Rodent models of PD can be classified into genetic and toxin-induced models. Genetic models carrying mutations in PD-associated genes such as *SNCA*, *PINK1*, *PARK2*, and *LRRK2* are better suited to study the progressive and hereditary aspects of the disease [57]. However, genetic models often fail to reproduce key features of human PD, including substantial dopaminergic neurodegeneration, authentic Lewy body formation, and the cardinal clinical motor symptoms [48,58,59]. In contrast, neurotoxin-induced models, including those induced by N-methyl-4-phenyl-l,2,3,6-tetrahydropyridine (MPTP), 6-hydroxydopamine (6-OHDA), or rotenone, selectively damage the nigrostriatal dopaminergic pathway, resulting in motor impairments such as bradykinesia, rigidity, and postural instability [42,60]. These neurotoxin-induced models are widely appreciated because of their reproducibility, rapid disease onset, cost efficiency, and neuropathological features such as oxidative stress, neuroinflammation, and Lewy body-like inclusions [42,60,61]. As summarized in Table 1, the rotenone model, by inhibiting mitochondrial complex I, induces progressive dopaminergic neurodegeneration, Lewy body-like inclusions, motor deficits, and non-motor symptoms such as gastrointestinal dysfunction and autonomic disturbances [62,63]. Owing to its ability to mimic both central and peripheral pathologies, the rotenone model serves as a valuable tool for investigating systemic PD mechanisms and MGBA interactions [62,63].

However, systemic administration of rotenone often results in high inter-individual variability and low reproducibility and requires prolonged exposure and intensive animal care [64]. The MPTP model produces rapid and reproducible dopaminergic neuron loss in the SN via its toxic metabolite MPP^+^, also targeting mitochondrial complex I [65]. It provides high reproducibility and is widely used to investigate the motor and non-motor symptoms of PD and mitochondrial dysfunction [65]. Nevertheless, the MPTP model rarely induces Lewy body formation, and its ability to reproduce behavioral features reminiscent of human PD remains limited [65]. In contrast, the 6-OHDA model, delivered by direct stereotaxic injection into specific brain regions, selectively destroys catecholaminergic neurons, resulting in robust and well-controlled dopaminergic lesions and motor impairments [42]. This model is suitable for evaluating neuroprotective interventions and cell replacement therapies. However, it lacks the Lewy body pathology, fails to replicate the progressive nature of human PD, and requires technically demanding surgical procedures [42].

Although no single model can fully recapitulate the complexity of human PD, these models, particularly rodent models, have collectively enhanced our understanding of both motor deficits and non-motor symptoms, including cognitive decline, anxiety, and gastrointestinal dysfunction (Table 1). Importantly, certain rodent models also display gut-related phenotypes that are particularly valuable for investigating the involvement of MGBA in PD pathophysiology and evaluating potential microbiota-targeted therapeutic interventions [66].

### 2.2. Multifaceted Neuroprotective Effects of Lactiplantibacillus plantarum in Rodent Models of Parkinson’s Disease

Multiple preclinical studies have demonstrated the neuroprotective potential of single-strain *Lpb. plantarum* in rodent models of PD, particularly in those induced by neurotoxins such as rotenone, MPTP, and 6-OHDA (Table 2). In rotenone-induced PD models, *Lpb. plantarum* PS128 (PS128, isolated from fermented vegetable) supplementation for four weeks significantly improved motor deficits (rotarod and narrow beam tests), increased striatal dopamine levels, preserved tyrosine hydroxylase (TH)-positive dopaminergic neurons, reduced microglial activation (ionized calcium binding adaptor molecule 1 and inducible nitric oxide synthase (iNOS), elevated brain-derived neurotrophic factor (BDNF)/tropomyosin receptor kinase B expression, and modulated inflammatory miR-155-5p/suppressor of cytokine signaling 1 (SOCS1) signaling [35]. Moreover, recent findings have demonstrated that PS128 supplementation markedly reduced α-syn aggregation in both the CNS and ENS, accompanied by increased serum levels of calcifediol and calcium, suggesting a potential calcium-regulatory mechanism contributing to its psychobiotic effects (unpublished results). Similarly, *Lpb. plantarum* CCFM405 (CCFM405, isolated from pickle samples) alleviated rotenone-induced motor impairments and improved performance in the rotarod, beam walking, and pole tests [36]. In addition to motor benefits, CCFM405 reversed gastrointestinal dysfunction, restoring colonic length, fecal output, fecal water content, attenuating colonic inflammation and epithelial damage by decreasing pro-inflammatory cytokines (interleukin-1β, interleukin-6, tumor necrosis factor-α), and restoring goblet cell populations [36]. Rotenone-induced dysbiosis was also partially reversed by *Lpb. plantarum* intervention, with an increased abundance of beneficial taxa, such as *Bifidobacterium*, *Faecalibaculum*, and *Turicibacter*, along with a reduced abundance of potentially pathogenic taxa, including *Alistipes* and *Ruminococcaceae* [35,36]. These microbial shifts suggest that *Lpb. plantarum* administration can effectively restore the gut microbial balance, which may contribute to slowing the progression of PD by modulating MGBA.

In MPTP-induced PD models, *Lpb. plantarum* DP189 (DP189, isolated from fermented sauerkraut) administration for 14 days significantly reduced α-syn aggregation in the SN, likely via suppression of oxidative stress, modulation of neuroinflammatory responses, and activation of the nuclear factor erythroid 2-related factor 2 (Nrf2)/antioxidant responsive element and peroxisome proliferator-activated receptor gamma coactivator 1-alpha (PGC-1α) signaling pathways, ultimately inhibiting NLR family pyrin domain containing 3 (NLRP3) inflammasome activation [37]. Similarly, *Lpb. plantarum* SG5, isolated from fermented mulberry leaves, reversed MPTP-induced α-syn accumulation in dopaminergic neurons [34]. In parallel, PS128 demonstrated robust neuroprotective effects by partially restoring striatal dopamine and 3,4-dihydroxyphenylacetic acid levels and preventing TH-positive neuron loss in both the SN and striatum. PS128 also inhibited glial activation (ionized calcium binding adaptor molecule 1 and glial fibrillary acidic protein), promotes neurotrophic factor expression (BDNF and nerve growth factor), and enhanced antioxidant defense through increased brain glutathione and superoxide dismutase activity [67]. DP189 supplementation further decreased apoptotic signaling by lowering the B-cell lymphoma 2 (Bcl-2) associated X protein/Bcl-2 ratio and Caspase-3 expression, whereas *Lpb. plantarum* CRL1905 (isolated from fermented amaranth sourdoughs) supplementation preserved TH-positive+ dopaminergic neurons and improved motor function [38,68].

In 6-OHDA-induced PD models that closely mimic the motor deficits observed in PD, PS128 induced marked neurobehavioral improvements. PS128 supplementation corrected aberrant cortical β-band oscillations, restored forelimb use symmetry, enhanced locomotor activity, and improved rotarod performance, achieving effects comparable to L-DOPA and deep brain stimulation [69]. Consistent findings have been reported for a probiotic mixture containing *Lpb. plantarum* LH05, *Limosilactobacillus fermentum* LH01, and *Limosilactobacillus reuteri* LH03, which were isolated from human milk and significantly reduced apomorphine-induced rotations and improved motor coordination [70]. Collectively, these results support the therapeutic potential of *Lpb. plantarum* in ameliorating the motor impairments associated with PD.

In addition to toxin-based models, metabolic dysregulation models have also demonstrated the neuroprotective potential of *Lpb. plantarum*. In high-fat diet-induced PD models, supplementation with a symbiotic formulation containing *Lpb. plantarum* DSM 20174 (isolated from pickled cabbage) significantly reduced hippocampal α-syn accumulation, oxidative stress, neuroinflammation, and neurotransmitter imbalance, suggesting broader metabolic neuroprotective capabilities [71]. Across these diverse PD models, *Lpb. plantarum* consistently demonstrated the ability to reduce α-syn pathology, attenuate oxidative stress and neuroinflammation, preserve dopaminergic neurons, improve motor and gastrointestinal functions, and modulate gut microbiota composition. These multifaceted effects support the potential of *Lpb. plantarum* as a promising gut-targeting agent for PD management.

## 3. Strain-Specific Clinical Efficacy of *Lactiplantibacillus plantarum* PS128 in Parkinson’s Disease

Currently, the clinical evidence supports the efficacy of a single-strain *Lpb. plantarum* against PD is limited. Several randomized controlled trials have reported significant improvements in bowel movement frequency, stool consistency, and constipation in PD patients using *Lpb. plantarum*-contained consortium. However, its effects on motor deficits remain unclear. These limited neurological benefits may be attributed to several factors, including strain-strain interactions that obscure individual probiotic effects, insufficient colonization due to competitive inhibition, or suboptimal strain selection for targeting neuroactive pathways. As summarized in Table 3, an 8-week trial using a high-dose multi-strain probiotic capsule showed improvements in gastrointestinal outcomes but failed to demonstrate changes in the Unified Parkinson’s Disease Rating Scale (UPDRS) motor scores [72]. Similarly, a 4-week intervention with a fermented milk product containing various strains and prebiotic fibers improved complete bowel movements but showed no benefit on dopaminergic symptomatology [73]. These findings suggest that multi-strain formulations may support general gut function, but they lack the specificity required to modulate neurological processes relevant to PD. Given these limitations, increasing attention has been focused on strain-specific psychobiotics.

A 2021 open-label clinical trial has demonstrated that PS128 supplementation (6 × 10^10^ CFU per day) for 12 weeks with constant anti-Parkinsonian medication significantly improved the motor score and quality of life of patients with PD [74]. In that study, clinical assessments included part III (UPDRS-III) motor scores, modified Hoehn and Yahr scale, and changes in ON-OFF diary recordings, which were designated as the primary outcome measures. Secondary outcomes were the 39-item Parkinson’s Disease Questionnaire (PDQ-39), Non-Motor Symptoms Scale, Beck Depression Inventory-II, Patient Assessment of Constipation Symptoms, Patient Global Impression of Change (PGI-C), and metabolic profiling of plasma and urine samples [74].

Compared with the baseline, PS128 supplementation resulted in significant improvements in motor performance. In the OFF state (the medication is not working well, and symptoms of PD have temporarily reappeared), UPDRS-III motor scores (−3.08 ± 4.41, *p* = 0.004) and akinesia subscores (−1.96 ± 3.01, *p* = 0.012) were significantly reduced. A trend toward a reduction in rigidity subscores (−0.60 ± 1.35, *p* = 0.057) was also observed. In the ON state, both motor scores (2.56 ± 5.36, *p* = 0.007) and total UPDRS scores (3.76 ± 6.04, *p* = 0.003) were also significantly improved [74]. PS128 administration shortened the OFF period by 48 min (*p* = 0.04) and extended the ON period by 50 min (*p* = 0.031) [74]. Although no significant improvements were observed in the overall non-motor symptom scores, this outcome may be attributable to the concurrent use of gastrointestinal medications during the PS128 intervention [74]. Nevertheless, meaningful improvements were detected in several subdomains of PDQ-39, including mobility, activities of daily living, stigma, and cognition [74]. Moreover, 68% of the participants reported improvement in PGI-C scores, suggesting that PS128 may contribute to improved health-related quality of life in patients with PD [74].

While most contemporary clinical studies on probiotics in PD focus on gastrointestinal symptom relief and employ multi-strain formulations, PS128 represents the first single-strain *Lpb. plantarum* intervention has been clinically shown to improve motor function in both the ON and OFF states, along with enhancing patient-reported quality of life [74]. These findings highlight the strain-specific potential of *Lpb. plantarum* as a promising adjunctive therapy for PD management.

## 4. Possible Molecular Mechanisms of *Lactiplantibacillus plantarum* on the Hallmark Pathologies of Parkinson’s Disease

### 4.1. Gut-Origin Hypothesis of α-Synuclein Pathology and Its Implications for Parkinson’s Disease Intervention

α-syn is a 14 kDa presynaptic protein encoded by the *SNCA* gene, predominantly expressed in neurons of both the CNS and ENS [75,76]. Under physiological conditions, α-syn is involved in the regulation of synaptic vesicle trafficking, dopamine release, and synaptic plasticity [75,76]. However, under pathological conditions, α-syn is highly susceptible to misfolding and aggregation [77]. This pathological transformation is driven by various genetic and stress-related factors. For example, mutations in the *SNCA* gene, such as A53T, A30P, and E46K, directly alter the conformation of the protein, enhancing its propensity to form toxic oligomers and fibrils [78].

In addition to *SNCA*, several other PD-related genes indirectly contribute to α-syn misfolding by disrupting cellular homeostasis [79]. Mutations in *PINK1* and *PARKIN* impair mitophagy, leading to mitochondrial dysfunction and increased production of reactive oxygen species (ROS), which promote protein oxidation and aggregation [80,81]. Deficiency in DJ-1, a redox-sensitive chaperone, further compromises cellular antioxidant defenses and increases vulnerability to oxidative stress [82,83]. Additionally, *LRRK2* mutations are associated with the hyperactivation of kinase signaling, interfering with vesicle trafficking and autophagic flux, both of which are essential for the clearance of misfolded proteins [84,85]. The mutants induce various forms of cellular stress, including oxidative stress, endoplasmic reticulum stress, and impaired autophagy, which in turn activate intracellular signaling cascades such as c-Jun N-terminal kinase, protein kinase R-like endoplasmic reticulum kinase-eukaryotic translation initiation factor 2 alpha subunit, and mammalian target of rapamycin [86]. These pathways facilitate protein misfolding and aggregation while impairing the degradation of toxic species [87,88]. As a result, α-syn transitions from its native soluble form into β-sheet-rich oligomers and insoluble fibrils, which progressively accumulate into Lewy bodies, triggering dopaminergic neuronal degeneration in the CNS, ultimately leading to PD progression [89].

While traditionally linked to the CNS, α-syn pathology is increasingly believed to originate from the ENS [90]. According to Braak’s hypothesis, α-syn misfolding may begin in the GI tract and ascend via retrograde transport through the vagus nerve to the medulla oblongata and brainstem nuclei, eventually reaching the SNpc [90,91]. This prion-like propagation mechanism is supported by clinical and experimental evidence. For instance, a study provided clinical evidence that the duodenum biopsies from PD patients contain forms of α-syn with self-propagating activity [92]. In animal models, the results also suggest that misfolded α-syn injection could induce the accumulation of endogenous α-syn in the GI tract and transmit it to the brain via the vagus nerve, providing mechanistic insight into the gut-to-brain spread of pathology [90,93]. Recent research has shown that enteroendocrine cells (EECs), which are specialized sensory epithelial cells in the gut lining, have many neuron-like characteristics and can produce endogenous α-syn [94]. α-syn expressed in EECs can undergo misfolding and aggregation and subsequently propagate to adjacent enteric neurons through direct cell-to-cell contact, thereby initiating the spread of pathology within the ENS [95]. This result suggests that EECs may be a potential initial site for peripheral α-syn pathology and a critical interface in gut–brain communication.

Taken together, these findings underscore the critical role of α-syn misfolding and aggregation in PD pathogenesis and gradually confirmed the ENS as a potential origin of this pathology. The prion-like propagation mechanism not only reinforces the gut-origin hypothesis but also suggests that early interventions targeting the GI tract may offer novel therapeutic opportunities. Therefore, increasing attention has been directed toward the gut microbiota and its modulation, particularly through psychobiotics interventions, such as *Lpb. plantarum*, being explored as a potential strategy to interfere with α-syn pathology.

### 4.2. Mechanisms of Lactiplantibacillus plantarum Against Parkinson’s Disease

As mentioned above, PD has increasingly been recognized as a disorder involving not only central neurodegeneration but also peripheral pathophysiological processes, particularly within the gut. Gut dysbiosis is considered a key contributing factor in PD pathogenesis, with emerging evidence suggesting that the misfolding and aggregation of gut-origin α-syn may precede and drive the progression of central pathology. Findings from both clinical and preclinical studies indicate that specific *Lpb. plantarum* strains are promising psychobiotics for the treatment of PD. However, the precise molecular mechanisms through which *Lpb. plantarum* affects PD remain largely unknown.

**Figure 2:**
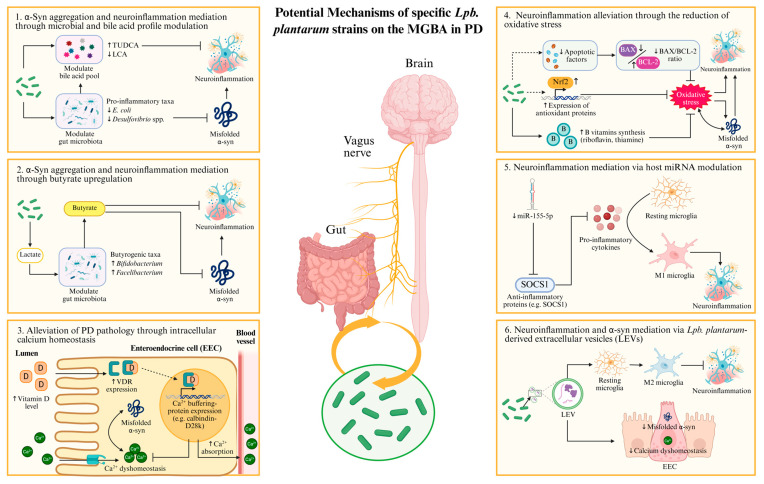
Mechanistic overview of *Lpb. plantarum* alleviates PD pathology via the microbiota-gut–brain axis. (**1**) *Lpb. plantarum* may indirectly attenuate neuroinflammation and α-syn aggregation via gut microbial profile and bile acid pool modulation. (**2**) *Lpb. plantarum* may indirectly upregulate butyrate levels through gut microbial modulation, which may in turn alleviate neuroinflammation and α-syn aggregation. (**3**) *Lpb. plantarum* may alleviate PD pathology via mediating impaired calcium absorption observed in PD. (**4**) *Lpb. plantarum* may alleviate oxidative stress via modulation of apoptotic factors, host antioxidant systems, and synthesis of antioxidants. (**5**) *Lpb. plantarum* may attenuate neuroinflammation via host miRNA modulation. (**6**) *Lpb. plantarum* may mediate neuroinflammation and α-syn pathology through the gut–brain axis via *Lpb. plantarum*-derived extracellular vesicles (LEVs). ↑, increased after intervention. ↓, decreased after intervention.

As shown in Figure 2, we highlighted and discussed several potential mechanisms through which *Lpb. plantarum* may exert neuroprotective effects in PD, with particular emphasis on its role in improving PD progression via MGBA.

#### 4.2.1. Modulation of Gut Microbiota and Bile Acid Signaling by *Lactiplantibacillus plantarum*: Implications for α-Synuclein Pathology

*Lpb. plantarum*, a lactic acid-producing bacterium with a large and flexible genome, is capable of rapid environmental adaptation through horizontal gene transfer. This property enables *Lpb. plantarum* to secrete diverse extracellular metabolites that may influence microbial ecology and host health [96,97]. A distinctive feature of *Lpb. plantarum* is its ability to produce bacteriocins, particularly plantaricins, which have been shown to inhibit the growth of Gram-negative pathogens by causing membrane disruption and cytoplasmic leakage [98]. The finding suggests that *Lpb. plantarum* may alleviate PD progression by reducing pathogen abundance. For example, in a PD-like mouse model, the relative abundance of *Escherichia coli* is significantly increased in the intestinal microbiota; in addition, *E. coli* administration triggered pathological α-syn accumulation in the colon and increased phosphorylation of α-syn caused by curli in *E. coli*-derived extracellular vesicles [99]. *Desulfovibrio* spp., its abundance is correlated with the severity of PD and has been implicated in promoting intestinal and systemic inflammation, including neuroinflammatory responses that may contribute to CNS damage and α-syn misfolding [100]. Importantly, scientific evidence indicates that supplementation with *Lpb. plantarum* can inhibit both *E. coli* and *Desulfovibrio* spp. [101,102]. Therefore, the suppression of these intestinal pathogens or pro-inflammatory bacteria by *Lpb. plantarum* may confer neuroprotective benefits via the modulation of MGBA.

In addition to its antimicrobial activity, *Lpb. plantarum* also plays a role in modulating bile acid (BA) metabolism. Some *Lpb. plantarum* strains, such as *Lpb. plantarum* ATCC14917 (isolated from cabbage), CF1 (isolated from sourdough), LT99 (isolated from raw-milk cheeses), and WCFS1 (isolated from human saliva), can express bile salt hydrolase (BSH), an enzyme that catalyzes the deconjugation of bile salts, thereby enhancing colonization under BA stress and selectively shaping BA pools toward receptors involved in BA homeostasis [103,104]. BAs, including primary and secondary two main types, is intricately linked to the composition of the gut microbiota and host neurophysiology [105,106]. BAs such as tauroursodeoxycholic acid (TUDCA) have demonstrated neuroprotective functions, including the inhibition of α-syn aggregation in the striatum of MPTP-induced PD model, while others, such as lithocholic acid (LCA) and deoxycholic acid, may exacerbate α-syn toxicity and inflammation [107,108]. Several studies have shown that specific *Lpb. plantarum* strains can beneficially modulate BA profiles by increasing the levels of neuroprotective TUDCA and reducing the levels of neurotoxic LCA [109,110,111,112]. For instance, *Lpb. plantarum* ATCC8014 can reduce LCA by elevating the relative abundances of *Allobaculum* and *Olsenella* [109]. Moreover, oral *Lpb. plantarum* strains can alter the BA profile by modulating the relative abundances of specific genera that play key roles in BA metabolism [112]. Collectively, these findings highlight the strain-specific activity of *Lpb. plantarum* to influence gut microbial ecology and BA metabolism, with potential downstream effects on α-syn pathology and neuroinflammation. This multifaceted interaction offers a promising mechanistic basis for psychobiotic applications in neurodegenerative disorders, such as PD.

#### 4.2.2. Butyrate-Mediated Modulation of α-Synuclein Aggregation and the Role of *Lactiplantibacillus plantarum* in Butyrogenesis

Butyrate, a four-carbon SCFA produced by gut microbiota through fermentation of dietary fibers, serves as a key energy source for colonocytes and plays a central role in maintaining intestinal and systemic homeostasis [113,114]. It contributes to the preservation of intestinal barrier integrity, modulates immune responses, and exerts both anti-inflammatory and neuroprotective effects [115,116]. By reinforcing intestinal barrier integrity, butyrate reduces systemic inflammation and peripheral endotoxin translocation, thereby attenuating neuroinflammatory responses [117,118]. Moreover, butyrate modulates neuroinflammation by suppressing microglial activation and downregulating pro-inflammatory mediators, including iNOS and nuclear factor-κB, resulting in decreased production of reactive oxygen and nitrogen species [119,120]

In addition to its anti-inflammatory effects, butyrate directly affects pathological protein aggregation and mitochondrial function in PD. In STC-1 cell models, butyrate promotes the degradation of misfolded α-syn by an autophagy-related protein 5-dependent and phosphatidylinositol 3-kinase/protein kinase B/mammalian target of rapamycin-related autophagy pathway [121]. In an MPTP-induced PD mouse model, butyrate supplementation protected against dopaminergic neuronal loss and motor dysfunction via stimulation of glucagon-like peptide 1 signaling [122]. Furthermore, recent studies have demonstrated that butyrate acts as a histone deacetylase inhibitor, promoting autophagy in rotenone-exposed dopaminergic neurons through epigenetic upregulation of PGC-1α, a critical regulator of mitochondrial function and defense against oxidative stress. This pathway ultimately leads to a reduction in α-syn expression [123].

Although *Lpb. plantarum* is not a direct butyrate producer; multiple studies have demonstrated its capacity to indirectly increase butyrate levels by promoting the growth of butyrogenic species via cross-feeding mechanisms [124,125]. For example, *Lpb. plantarum* produces lactate, a metabolic intermediate that can be converted to pyruvate and utilized in the butyrate biosynthesis pathway [126]. Co-culture studies have shown that lactate produced by *Bifidobacterium* may serve as a substrate for butyrate-producing microbes, such as *Megasphaera indica*, while synergistic cross-feeding between *Bifidobacterium adolescentis* and *Faecalibacterium prausnitzii* enhances butyrate production [127,128]. Furthermore, probiotic supplementation with *Lpb. plantarum* 16 and *Paenibacillus polymyxa* 10 have been shown to increase *F. prausnitzii* abundance and improve the intestinal epithelial barrier function in animal models [129]. Collectively, these findings suggest that *Lpb. plantarum* may exert indirect butyrogenic effects by modulating the microbial networks that support butyrate-producing species, potentially contributing to α-syn clearance and reducing neuroinflammation.

#### 4.2.3. Probiotic Modulation of Intracellular Calcium Homeostasis as a Strategy to Alleviate α-Synuclein Pathology in Parkinson’s Disease

Calcium dysregulation plays a central role in PD pathogenesis by promoting α-syn aggregation. As mentioned above, α-syn is a presynaptic protein involved in synaptic transmission. It can bind calcium and elevate intracellular calcium levels, facilitating its misfolding and oligomerization. α-syn aggregation ultimately leads to Lewy body formation and dopaminergic neurodegeneration. Neurologically, dopaminergic neurons in the SNpc are susceptible to calcium dysregulation due to the high energy demands required to maintain pacemaker activity and mitochondrial function [130,131]. Disruptions in calcium handling or mitochondrial function significantly increase neuronal vulnerability to oxidative stress [132]. In turn, oxidative stress and calcium dysregulation synergistically exacerbate α-syn misfolding, oligomerization, and fibril formation, thereby establishing a self-perpetuating feedback loop that further impairs mitochondrial integrity and accelerates neurodegeneration [133].

Although direct evidence linking *Lpb. plantarum* to the regulation of calcium homeostasis in PD remains limited, findings from related studies offer valuable insights into its potential neuroprotective mechanisms. Specific *Lpb. plantarum* strains have been reported to enhance or promote vitamin D biosynthesis in the host [134]. Vitamin D3 exerts neuroprotective effects by upregulating the glial cell line-derived neurotrophic factor, which is critically involved in the survival and maintenance of dopaminergic neurons [135]. In addition to its neurotrophic effects, vitamin D plays an essential role in calcium homeostasis and may interfere with α-syn aggregation at early stages, as evidenced by reduced oligomer formation in SH-SY5Y neuronal cells following vitamin D treatment [136]. Moreover, vitamin D analogs, such as calcipotriol, have demonstrated the ability to induce calcium-buffering proteins, including calbindin-D28k, which effectively attenuate calcium overload and suppress α-syn aggregation under oxidative conditions [137]. Elevated levels of calbindin-D28k in SNpc neurons are correlated with reduced apoptotic susceptibility, further supporting the neuroprotective role of calcium-buffering mechanisms in dopaminergic neurons [138].

Recently, preliminary investigations suggested that PS128 may regulate α-syn aggregation through vitamin D-dependent calcium homeostasis pathways. In rotenone-induced PD-like mice, PS128 treatment significantly restored serum calcium and 25-hydroxyvitamin D levels, upregulated intestinal vitamin D receptor protein expression, and stimulated calcium transport, contributing to improved systemic calcium handling (unpublished results).

Collectively, these findings support a mechanistic framework whereby *Lpb. plantarum* enhances host vitamin D bioavailability and activates calcium-buffering systems, thereby attenuating intracellular α-syn aggregation and providing dopaminergic neuroprotection. Modulation of this gut microbiota-vitamin D-calcium-regulatory axis may offer a promising therapeutic approach targeting MGBA in PD.

#### 4.2.4. *Lactiplantibacillus plantarum*-Mediated Regulation of Oxidative Stress in Parkinson’s Disease

Oxidative stress is a key contributor to the pathogenesis of PD, where excessive production of ROS disrupts redox homeostasis, leading to dopaminergic neuronal loss and α-syn aggregation [139]. Recent studies have suggested that *Lpb. plantarum* protects neuronal cells against oxidative insult by modulating apoptotic pathways. For instance, *Lpb. plantarum* KU210152 (isolated from kimchi) reduced ROS generation, suppressed caspase-3 and caspase-9 activity, and decreased the Bcl-2-associated X/Bcl-2 ratio in H_2_O_2_-challenged SH-SY5Y cells, conferring neuroprotection [140]. Similarly, *Lpb. plantarum* 200655 (isolated from kimchi) exerted comparable anti-apoptotic effects in oxidative stress-induced SH-SY5Y cells [141,142]. Moreover, *Lpb. plantarum*-fermented herbal formula SGT166 (Sagunja-tang) significantly inhibited toxin-induced intracellular H_2_O_2_ accumulation, preserved mitochondrial membrane potential, and attenuated mitochondria-mediated apoptosis, whereas its unfermented counterpart lacked these protective effects [143].

In addition to direct ROS scavenging, *Lpb. plantarum* can activate the host antioxidant defense systems. Nrf2, a master regulator of oxidative stress, has been shown to be activated by multiple *Lpb. plantarum* strains, leading to elevated antioxidant enzyme activities and suppression of neuroinflammatory mediators such as NLRP3 inflammasome [144,145,146,147]. In the MPTP-induced PD mouse model, DP189 administration significantly reduces α-syn accumulation in the SN via activating Nrf2 signaling [37]. Furthermore, Nrf2 activation correlates with increased BDNF expression. *Lpb. plantarum* may offer neuroprotective benefits, potentially protecting against neurodegenerative diseases or cognitive decline [148].

Another potential antioxidant mechanism is the *Lpb. plantarum*-mediated vitamin B biosynthesis. Specific strains such as *Lpb. plantarum* CRL1905 and CRL2130 have demonstrated the ability to synthesize thiamine (vitamin B1) and riboflavin (vitamin B2), respectively, both of which attenuated ROS elevation [149,150]. Genomic analyses revealed the presence of rib operon-associated riboflavin biosynthesis genes in many *Lpb. plantarum* strains [151]. Collectively, these findings highlight the multifaceted antioxidative capacity of *Lpb. plantarum*, which may protect against mitochondrial oxidative injury, reduce α-syn aggregation, and ultimately attenuate neurodegeneration, underscoring its promise as a psychobiotic candidate for PD intervention.

#### 4.2.5. Involvement of MicroRNAs in *Lactiplantibacillus plantarum*-Mediated Attenuation of Parkinsonian Pathology

MicroRNAs (miRNAs) are small non-coding RNA molecules (approximately 18–24 nucleotides) that modulate gene expression at the post-transcriptional level by binding to the 3‘ untranslated regions (3’-UTRs) of target mRNAs, thereby repressing translation [152,153,154]. Some studies have suggested that miRNAs can regulate α-syn expression by targeting its mRNA, thereby modulating Lewy body pathology [155]. Specifically, miR-7, miR-153, miR-34b, miR-34c, miR-214, and miR-1643 have been identified to directly bind to the 3’-UTR of α-syn mRNA and downregulate its expression. These miRNAs can also indirectly impact α-syn levels by targeting other genes that influence its degradation or accumulation [155,156,157,158,159].

Although direct evidence linking *Lpb. plantarum* to the regulation of α-syn via miRNAs is currently lacking; existing data suggest that *Lpb. plantarum* may alleviate PD-related pathology by modulating inflammatory responses. Lee et al. (2023) have demonstrated that PS128 modulated miR-155-5p expression to exert immunoregulatory effects in a rotenone-induced PD mouse model [35]. miR-155-5p is a pro-inflammatory miRNA that promotes M1 microglial polarization and contributes to neuroinflammation, a key factor in PD pathogenesis [160]. Mechanistically, miR-155-5p directly targets the 3’-UTR of SOCS1 mRNA, inhibiting its translation and thereby enhancing inflammatory signaling [161]. PS128 administration significantly reduced the levels of miR-155-5p and increased the expression of SOCS1 in the brains of PD-like mice [35]. Importantly, miR-155-5p reduction is positively correlated with motor deficits in PD-like mice [35]. Collectively, these findings suggest that *Lpb. plantarum* may reduce neuroinflammation by regulating the miR-155-5p-SOCS1 axis, thereby alleviating PD progression.

## 5. *Lactiplantibacillus plantarum*-Derived Extracellular Vesicles: A Novel Mediator in Microbiota-Gut–Brain Axis and α-Synuclein Pathology

In recent years, postbiotics derived from live microorganisms have gained increasing attention in the field of microbe-host interactions. Multiple strains of *Lpb. plantarum* exhibit the ability to secrete extracellular vesicles (EVs) that encapsulate SCFAs, proteins, and metabolites [162]. *Lpb. plantarum*-derived EVs (LEVs) can cross the BBB and be internalized by host cells via receptor-mediated phagocytosis, direct membrane fusion, or endocytosis [163,164]. Once internalized, LEV cargos may interact with host immune receptors such as Toll-like receptor 2, subsequently activating downstream nuclear factor-κB signaling pathways and modulating immune cell phenotypes [162]. Furthermore, emerging evidence indicates that LEVs contain small RNAs that are functionally similar to host miRNAs, which may integrate into the human genome and exert gene-regulatory effects [165].

Recent studies have demonstrated that LEVs can drive monocytes toward an anti-inflammatory M2 macrophage phenotype, accompanied by elevated secretion of interleukin-10 [166,167]. In neuronal cells, LEVs have been shown to upregulate BDNF expression, and neurotrophins are inversely correlated with α-syn expression [168,169,170]. Additionally, LEVs have been reported to reduce neuronal apoptosis in ischemic stroke models through miR-101a-3p-mediated mechanisms [171]. Although substantial evidence supports LEV-induced M2 macrophage polarization, the regulatory roles of LEV in CNS glial cells, such as microglia and astrocytes, remain largely unexplored. Given their capacity to cross the BBB, it has been hypothesized that *Lpb. plantarum* alleviates neuroinflammation by secreting LEVs that promote microglial M2 polarization.

EECs, which constitute a key interface between the gut epithelium and luminal microbiota, have been proposed as an initial site for α-syn aggregation in the gut [94]. Although direct evidence for microbial EV uptake by EECs is currently lacking, this possibility has attracted increasing scientific interest. For example, milk-derived EVs have been shown to enter EECs and disrupt α-syn homeostasis via the miRNA-148a/DNMT1 pathway, as well as interfere with autophagy-related miRNA-148a/PPARGC1A and miRNA-21/LAMP2A axes [172]. Moreover, *Akkermansia muciniphila*, which is frequently enriched in the gut microbiota of patients with PD, has been demonstrated that its culture medium induces mitochondrial calcium imbalance in EECs, thereby promoting α-syn aggregation [173]. This calcium imbalance is speculated to be mediated by *A. muciniphila*-derived metabolites, secreted factors, or EVs that interfere with the intracellular calcium regulation. Based on these findings, it is speculated that *Lpb. plantarum* may mitigate α-syn aggregation and its pathological progression in EECs through EV-mediated mechanisms.

## 6. Future Challenges and Conclusions

In recent decades, gut-targeted therapies for PD have shown promise, with several *Lpb. plantarum* strains demonstrate dopaminergic restoration and symptom alleviation in preclinical models. However, the translation of these findings into clinical applications remains challenging. For example, owing to the invasive procedures hindering patient recruitment, most clinical trials focus primarily on improving gastrointestinal symptoms, with limited evaluation of CNS biomarkers such as dopamine or α-syn. Therefore, the development of minimally invasive biomarker strategies is critical to advance our mechanistic understanding of psychobiotics in human PD.

Disease heterogeneity is another challenge in the development of effective psychobiotic therapies for PD. Previous studies have demonstrated that gut- and brain-origin PD subtypes exhibit distinct patterns of dysbiosis, suggesting that standardized probiotic interventions may not be universally effective. Additionally, individual differences in gut microbiota composition, influenced by factors such as age, ethnicity, dietary habits, and medication exposure, have been linked to variable clinical responses. These findings suggest the importance of precision-based approaches that tailor probiotic formulations to the microbial and clinical profiles of patients. The application of multi-omics technologies, including metagenomics, proteomics, and metabolomics, may support the identification of predictive biomarkers and strain-specific mechanisms, thereby enhancing the consistency and efficacy of psychobiotic interventions in diverse PD populations.

Building on these insights, advancing *Lpb. plantarum* as a psychobiotic strategy for PD will require a deliberate balance between mechanistic depth and clinical applicability. Although multi-strain probiotic formulations have dominated PD research, accumulating evidence indicates that certain single strains, such as PS128, may exert neuroprotective benefits through gut microbial modulation, enhancement of intestinal barrier integrity, regulation of calcium homeostasis, and attenuation of neuroinflammation. Nevertheless, the existing clinical evidence is limited and is often derived from small-scale, open-label studies with heterogeneous patient cohorts, variable dosing regimens, and short intervention durations. To bridge the gap between mechanistic discovery and therapeutic implementation, a stepwise translational framework is necessary, beginning with the systematic characterization of strains in preclinical models, followed by early-phase clinical trials that integrate MGBA-related biomarkers, and culminating in large-scale, randomized, double-blind, placebo-controlled studies powered for both motor and non-motor outcomes. Incorporating biomarker-based monitoring, long-term safety assessments, and personalized intervention strategies is essential for addressing disease heterogeneity and inter-individual microbiota variation. Ultimately, the successful clinical translation of *Lpb. plantarum* as a psychobiotic for PD will depend on the integration of mechanistic validation with robust clinical evidence to establish strain-specific efficacy and safety profiles.

## Figures and Tables

**Table 1:** Comparisons of the neurotoxin-induced rodent models of PD.

	Rotenone	MPTP	6-OHDA
Primary mechanism	Mitochondrial Complex I inhibition	Mitochondrial Complex I inhibition	Selective degeneration of catecholaminergic neurons
Site of action	Systemic (brain and gut)	SNpc	Nigrostriatal pathway (injected site)
Rodent species used	Rat and mouse	Mouse	Rat
Onset and duration	Chronic (weeks to months)	Acute/subacute (days-weeks)	Acute (1–2 weeks)
Lewy body presence	Yes	Absent	Absent
Motor deficit	Moderate-high	High	Very high
Non-motor symptom	High	High	Limited
MGBA relevance	High	Moderate	Limited
Model variability	High	Low	Low
Experimental cost	Hight	Moderate	Moderate-high
References	[60,62,63,64]	[60,65]	[42,60]

SNpc, substantia nigra pars compacta; MPTP, N-methyl-4-phenyl-l,2,3,6-tetrahydropyridine; 6-OHDA, 6-hydroxydopamine.

**Table 2:** Summary of single-strain *Lpb. plantarum* studies on PD models.

Treatment	Model	Dosage (CFU)	Period	Sample Size	Main Findings	Ref.
*Lpb. plantarum* PS128	Rotenone	1 × 10^9^	6 weeks	4 groups; *n* = 10 per group	Improved motor function [↓Walking time on NBT, ↑Retention time on RTR] Neuroprotection [↑TH+, ↓Iba1, ↑BDNF, ↑DA] Microbiota modulation [↑*Bifidobacterium*, ↓*Ruminococcaceae*_UCG_014, ↓*Bacteroides*, ↓*Alistipes*]	[35]
*Lpb. plantarum* CCFM405	Rotenone	1 × 10^9^	8 weeks	3 groups; *n* = 12 per group	Neuroprotection [↑TH+ in STR, ↑DA, ↑5-HT, ↓Microglia activation, ↓Astrocyte activation] Improved motor function [↓Time on PT, ↓Walking time on NBT, ↑Retention time on RTR, ↑Total walking distance in OFT] Reduced GI deficits [↑Colon length, ↑Fecal pellet size, ↑Intestinal lining thickness (↑ZO-1, ↑Occludin), ↑Goblet cell count] Reduced intestinal inflammation [↓IL-6, ↓TNF-α] Microbiota modulation [↑*Bifidobacterium*, ↑*Faecalibaculum,* ↑*Turicibacter*, ↓*Alistipes*, ↓*Akkermansia*, ↓*Bilophila*, ↓*Ruminococcaceae*_UCG_004, ↓*Ruminococcaceae*_UCG_009] Altered serum and fecal metabolite composition	[36]
*Lpb. plantarum* DP189	MPTP	2 × 10^8^	2 weeks	4 groups; *n* = 10 per group	Reduced α-syn aggregation Neuroprotection [↑SOD, ↑GSH-Px, ↑IL-10, ↓MDA, ↓ROS, ↓TNF-α, ↓IL-6, ↓IL-1β] Microbiota modulation [↑*Prevotella*, *↓Proteobacteria*, *↓Actinobacteria*]	[37]
*Lpb. plantarum* SG5	MPTP	1 × 10^9^	5 weeks	5 groups; *n* = 10 per group	Reduced α-syn aggregation Neuroprotection Improved motor function Microbiota modulation [↑*Bacteroidetes*, ↑*Proteobacteria*, *↓Desulfovibrio*]	[34]
*Lpb. plantarum* PS128	MPTP	1 × 10^9^	4 weeks	4 groups; *n* = 18 per group	Neuroprotection [↑DA, ↑DOPAC, ↑TH+, ↑BDNF, ↑NGF, ↓Iba1, ↓GFAP, ↓TNF-α/IL-1β/IL-6] Improved motor function [↓Inversion time and descent time on PT, ↓Walking time on NBT, ↑Retention time on RTR] Microbiota modulation [↓*Enterobacteriaceae*] ↓Oxidative stress [↑GSH, ↑SOD]	[67]
*Lpb. plantarum* CRL1905	MPTP	8 ± 2 × 10^8^	4 weeks	5 groups; *n* = 6 per group	Neuroprotection [↑TH+, ↓IL-6, ↓TNF-α, ↓IFN-γ, ↓MCP-1] Improved motor function Increased thiamine production	[68]
*Lpb. plantarum* PS128	6-OHDA	1.5 × 10^10^	12 weeks	L-dopa *n* = 7 DBS *n* = 6 PS128 *n* = 9 Saline *n* = 4	Neuroprotection [↑DA, ↓turnover ratios of DA and NA, ↑TH+] Improved motor function [↓β-PSD, ↑Contralateral paw use, ↑Total walking distance in OFT]	[69]

BDNF, brain-derived neurotrophic factor. DA, dopamine. DOPAC, 3,4-dihydroxyphenylacetic acid. GFAP, glial fibrillary acidic protein. GSH-Px, glutathione peroxide. Iba1, ionized calcium binding adaptor 1. IFN-γ, interferon gamma. IL-1β, interleukin 1 beta. IL-6, interleukin 6. IL-10, interleukin 10. MCP-1, monocyte chemoattractant protein-1. MDA, malondialdehyde. NA, noradrenaline. NBT, narrow beam test. NGF, nerve growth factor. OFT, open field test. PT, pole test. ROS, reactive oxygen species. RTR, rotarod test. SOD, superoxide dismutase. STR, striatum. TH, tyrosine hydroxylase. TNF-α, tumor necrosis factor alpha. ZO-1, zonula occludens-1. 5-HT, 5-hydroxytryptamine. β-PSD, power spectral density of beta oscillations. ↑, increased after intervention. ↓, decreased after intervention.

**Table 3:** Summary of probiotic interventions containing *Lpb. plantarum* in PD patients.

Treatment	Dosage (CFU)	Period	Sample Size	Main Findings	Ref.
*Lpb. plantarum*, *Lbs. casei*, *Lab. acidophilus*, *Lab. bulgaricus*, *B. infantis*, *B. longum*, *B. breve*, *S. thermophilus*	4.5 × 10^11^	8 weeks	2 groups; Placebo (*n* = 13) Probiotics (*n* = 14)	Improved gastrointestinal outcomes [↑Number of defecations with a sense of complete evacuation per week, ↑Stool consistency, ↑Frequency of bowel movements]	[72]
*S. thermophilus*, *E. faecium*, *Lbs. rhamnosus* GG, *Lab. acidophilus*, *Lpb. plantarum*, *Lbs. paracasei*, *Lab. bulgaricus*, *B. breve*, *B. lactis* and prebiotic fibers	2.5 × 10^11^	4 weeks	2 groups; Placebo (*n* = 40) Probiotics (*n* = 80)	Improved gastrointestinal outcomes [↑Number of complete bowel movements, ↑Stool consistency, ↓Use of laxatives]	[73]
*Lpb. plantarum* PS128	6 × 10^10^	12 weeks	1 group Probiotics (*n* = 25)	Improved motor function [OFF state (↓UPDRS-III motor scores, ↓akinesia subscores), ↓OFF period, ON state (↓UPDRS-III motor scores, ↓Total UPDRS scores), ↑ON period] Improved quality of life [Mobility, Activities of daily living, Stigma, Cognition, PGI-C]	[74]

↑, increased after intervention. ↓, decreased after intervention.

## Data Availability

Data are contained in the article and cited articles.

## Abbreviations

The following abbreviations are used in this manuscript:

5-HT	5-hydroxytryptamine
6-OHDA	6-hydroxydopamine
BA	Bile acid
BBB	Blood–brain barrier
Bcl-2	B-cell lymphoma 2
BDNF	Brain-derived neurotrophic factor
BSH	Bile salt hydrolase
CNS	Central nervous system
DA	Dopamine
DOPAC	3,4-dihydroxyphenylacetic acid
EEC	Enteroendocrine cells
ENS	Enteric nervous system
EV	Extracellular vesicles
GFAP	Glial fibrillary acidic protein
GI	Gastrointestinal
GSH	Glutathione peroxide
Iba1	Ionized calcium-binding adaptor molecule 1
IFN-γ	Interferon-gamma
IL-1β	Interleukin-1 beta
IL-6	Interleukin-6
IL-10	Interleukin-10
iNOS	Inducible nitric oxide synthase
LCA	Lithocholic acid
L-DOPA	Levodopa
LEVs	*Lactiplantibacillus plantarum*-derived EVs
MCP-1	Monocyte chemoattractant protein-1
MDA	Malondialdehyde
MGBA	Microbiota-gut–brain axis
miRNA	Micro RNAs
MPTP	N-methyl-4-phenyl-l,2,3,6-tetrahydropyridine
NA	Noradrenaline
NBT	Narrow beam test
NGF	Nerve growth factor
NLRP3	NLR family pyrin domain containing 3
Nrf2	Nuclear factor erythroid 2-related factor 2
OFT	Open field test
PD	Parkinson’s disease
PDQ39	39-item Parkinson’s Disease Questionnaire
PGC-1α	Peroxisome proliferator-activated receptor gamma coactivator 1-alpha
PGI-C	Patient Global Impression of Change
ROS	Reactive oxygen species
RTR	Rotarod test
SCFA	Short-chain fatty acid
SNpc	Substantia nigra pars compacta
SOCS1	Suppressor of cytokine signaling 1
SOD	Superoxide dismutase
STR	Striatum
TH	Tyrosine hydroxylase
TNF-α	Tumor necrosis factor-alpha
TUDCA	Tauroursodeoxycholic acid
UPDRS	Unified Parkinson’s Disease Rating Scale
PT	Pole test
ZO-1	Zonula occludens-1
α-syn	Alpha-synuclein
β-PSD	Power spectral density of beta oscillations
